# Different Responses of Microbiota across Intestinal Tract to *Enterococcus faecium* HDRsEf1 and Their Correlation with Inflammation in Weaned Piglets

**DOI:** 10.3390/microorganisms9081767

**Published:** 2021-08-19

**Authors:** Jin Zhou, Ji Luo, Shumin Yang, Qiling Xiao, Xiliang Wang, Zutao Zhou, Yuncai Xiao, Deshi Shi

**Affiliations:** State Key Laboratory of Agriculture Microbiology, College of Veterinary Medicine, Huazhong Agricultural University, Wuhan 430070, China; jinzhou@webmail.hzau.edu.cn (J.Z.); xifengcoo@163.com (J.L.); yang2021095@163.com (S.Y.); xiaoqilingk@sina.com (Q.X.); wxl070@mail.hzau.edu.cn (X.W.); ztzhou@mail.hzau.edu.cn (Z.Z.)

**Keywords:** *Enterococcus faecium* HDRsEf1, weaned piglets, gut microbiota, gut inches, inflammation, butyrate-producing bacteria

## Abstract

*Enterococcus faecium* HDRsEf1 (HDRsEf1) was identified to reduce the incidence of diarrhea in weaned piglets, but the mechanism has not been elucidated yet. Based on the fact that gut microbiota plays a crucial role in regulating inflammatory responses, the effects of HDRsEf1 on microbiota across the intestinal tract in weaned piglets were investigated. Microbiota from the luminal contents and the mucosa of the ileum, cecum, and colon of HDRsEf1-treated piglets were explored by 16S rRNA sequencing and qPCR. It was demonstrated that microbiota in different gut niches responded specifically to HDRsEf1, with major alterations occurring in the ileum and cecum. The total bacterial load of microbiota in ileal luminal contents and the relative abundance of *Escherichia-Shigella* in the ileal mucosa was significantly down-regulated by HDRsEf1 administration, while the relative abundance of butyrate-producing bacteria (including *Clostridiaceae-1*, *Rumencoccidae*, and *Erysipelotrichaceae*) in cecal luminal contents was significantly up-regulated. Moreover, the utilization of HDRsEf1 improved intestinal morphological development and reduced the inflammatory response, which were negatively correlated with the relative abundance of *Escherichia-Shigella* in the ileal mucosa and butyrate-producing bacteria in cecal luminal contents, respectively. Collectively, this study suggests that the administration of HDRsEf1 alters gut microbiota, thereby alleviating inflammation and improving intestinal morphological development in weaned piglets.

## 1. Introduction

In the pig rearing industry, early weaning stress of piglets often leads to an increased diarrhea rate and growth retardation [1]. Since the 1850s, a subclinical dose of antibiotics has been used in animal husbandry, especially in piglet breeding, to reduce the diarrhea rate and promote the growth of piglets [2]. However, the utilization of antibiotics as feed additives has caused the emergence and spread of drug-resistant bacteria and antibiotic residues in pig meat. That is why antibiotic consumption for pig production has been gradually banned in several countries [3]. Ever since the ban on antibiotic as growth promoters in animal production, the application of probiotics as alternatives to antibiotics has progressively started [4]. *E. faecium* is one of the first batch of probiotics approved by the EU and FDA for animal feed [5]. Our previous studies have shown that HDRsEf1 could promote growth and reduce the incidence of diarrhea in piglets; however, the protective mechanism still needs to be investigated [6]. Many studies reported that *E. faecium* exhibited several beneficial functions, including enhancing the intestinal barrier integrity, inhibiting pathogen adhesion and infection, inducing anti-inflammatory and anti-oxidant effects, and promoting immune system development [7,8,9,10,11,12]. However, the effects of *E. faecium* on gut microbiota have not been investigated yet. A variety of microorganisms inhabit in the gastrointestinal tract of mammals and have profound influences on host development, nutrient digestion, and immune modulation [13,14,15]. In early life, multiple factors, such as diet, therapeutic agents, and infection shape the host’s initial gut microbiota, which, consequently, cause a long-term impact on the host [16]. It is well known that antibiotics promote growth by affecting the number and structure of the microbiota in the small intestine [17,18], and some probiotics can also promote animal growth and improve health by regulating gut microbiota of piglets [19,20]. *E. faecium* belongs to lactic acid bacteria, and some strains can secrete organic acids, bacteriocin, and other antibacterial substances [21]. In addition, HDRsEf1, which belongs to *E. faecium,* has probiotic effects on different animals, including mammals (pigs) and poultry (broilers) [6]. Therefore, it is reasonable to hypothesize that the effects of HDRsEf1 on piglets may be due to its regulation on gut microbiota.

There are different microenvironments along the whole gastrointestinal tract, which contribute to spatial changes in the bacterial composition [22,23,24,25]. Studies have shown microbiota in different gut niches respond differently to dietary changes [26,27]. Although many previous studies have explored the impact of probiotics on gut microbiota, due to the convenience of sampling, previous studies mostly took fecal and colonic contents as samples, ignoring the regional differences of gut microbiota.

Therefore, this study was designed to explore the effects of HDRsEf1 on microbiota in different gut niches, including luminal contents and the mucosa of the ileum, cecum, and colon. Moreover, given that weaning stress could cause excessive inflammation, which results in intestinal injury and diarrhea, the correlation between changes in microbial composition and inflammation or intestinal development were also assessed [28].

## 2. Materials and Methods

### 2.1. Animal Husbandry

Animal experiment was carried out at the experimental station of Huazhong Agricultural University in Hubei Province, China. All animal protocols used in this study were in accordance with the Guidelines for the Care and Use of Animals for Research and Teaching and approved by the Animal Care and Use Committee of Huazhong Agricultural University (permit number: HZAUSW2018-018). Three-week-old male (Landrace × Large White) post-weaning piglets with similar body weights were assigned to HDRsEf1 treatment group or control group (n = 5). All the piglets were weaned on the 21st day of age and had an adaption period for one week. After adaption, the piglets were randomly allocated into two groups: a control group and the HDRsEf1 treatment group. The control group was administered with the basal diet according to nutrient requirements (NRC, 2012). The HDRsEf1 treatment group was administered the basal diet supplemented with *E. faecium* HDRsEf1 at 5 × 10^6^ CFU/g for 28 days [6]. The HDRsEf1 was provided by Huada Ruier Co., Ltd. (Wuhan, China). All piglets were free to feed and drink, weighed once a week, and no antibiotics were used during the experiment.

### 2.2. Slaughtering and Sampling

After fasting overnight, at the 28th day of HDRsEf1 treatment, piglets were sacrificed for samples collection. Ileum, cecum, and middle colon were ligated, and then the luminal contents and mucosal tissue were collected from these sites. After being washed with PBS, the mucosal tissue was scraped by a glass slide. All samples were stored at −80 °C after being quick frozen with liquid nitrogen. For the measurements of serum inflammatory factors, the serum samples from these sacrificed piglets were collected. The duodenum, jejunum, and ileum tissues were collected and immediately fixed in 4% paraformaldehyde for subsequent morphological analysis. 

### 2.3. DNA Extraction, 16S rRNA Gene Amplification, and High-Through Sequencing

A total of 0.5 g lumen contents and mucosal scraps of ileum, cecum, and colon were used to extract total bacterial genomic DNA using the QIAamp R Fast DNA Stool Mini Kit (QIAGEN Ltd., Hilden, Germany) according to the manufacturer’s protocol. The V3–V4 region of the 16S rRNA gene was amplified using universal primers 338F (5′-ACTCCTACGGGAGGCAGCAG-3′) and 806R (5′-GGACTACHVGGGTWTCTAAT-3′). The PCR reactions were conducted using the following program: denaturation at 95 °C for 3 min, 27 cycles of 95 °C for 30 s, annealing at 55 °C for 30 s, elongation at 72 °C for 45 s, and a final extension at 72 °C for 10 min. The PCR components, the extraction and purification of PCR products were the same with previous study [29]. Purified PCR products were pooled into equimolar amounts and sequenced on the Illumina MiSeq platform according to the standard protocols by Shanghai Majorbio Bio-pharm Technology Co., Ltd. (Shanghai, China). The raw reads were deposited into the NCBI Sequence Read Archive (SRA) database (Accession Number: PRJNA694358).

### 2.4. Analysis of Sequencing Data

Raw FASTQ files were demultiplexed, quality-filtered by Trimmomatic, and merged by FLASH by the criteria described previously [30]. Operational taxonomic units (OTUs) were clustered with 97% similarity cutoff using UPARSE (version 7.1 http://drive5.com/uparse/, accessed on 26 April 2020) and chimeric sequences were identified and removed using UCHIME [31]. The taxonomy of each 16S rRNA gene sequence was analyzed by RDP Classifier algorithm (http://rdp.cme.msu.edu/, accessed on 26 April 2020) against the Silva (silva 132/16s bacteria) database using confidence threshold of 70% [32].

Sob and Shannon indices were used to reflect α diversity at 97% identity and plotted using Mothur (version v.1.30.2) [33]. In β diversity analysis, unweighted principal coordinates and weighted principal coordinates based on the abundance of OTU were used to determine the difference of microbiota between groups. Analysis of similarities (ANOSIM) was performed to assess the overall similarity between groups by testing the significance of spatial separation in PCoA using the “vegan” package [34]. According to the composition and sequence distribution of samples at each taxonomic level, the significant differential abundant genera between groups were tested by Student’s *t*-test and visualized using the R package software (version 3.3.1) [35]. Linear discriminant analysis (LDA, threshold set to 2 or 3) effect size (LEfSe) was used to elucidate the differences of bacterial taxa. The algorithm uses the nonparametric factorial Kruskal–Wallis sum rank test to detect features with significant differential abundance, followed by linear discriminant analysis to estimate the effect size of each differentially abundant taxa. The cladogram was drawn using the LEfSe algorithm (http://huttenhower.sph.harvard.edu/lefse/, accessed on 27 November 2020) [36]. The correlations between bacterial composition and inflammatory cytokine expression or the development of intestinal villi were calculated by spearman correlation coefficient and displayed by the heatmap generated by R (version 3.3.1) using the “pheatmap” package [37].

### 2.5. RNA Extraction and RT-qPCR of Small Intestine

Total RNA was extracted from duodenum, jejunum, and ileum tissues using Trizol reagent (Takara Bio, Otsu, Japan) and quantified for cDNA synthesis. Total RNA (1 μg) was reverse-transcribed using a PrimeScript^®^ RT reagent Kit with gDNA Eraser (Takara Bio, Otsu, Japan), carefully following the manufacturer’s instructions. The primers and amplification conditions for inflammatory cytokines (IL-1β, IL-8, IL-12p35, IL-12p40, TNF-α, and IFN-γ), NFκB, and GAPDH are listed in Table S1. Quantitative real-time PCR (RT- qPCR) was performed using SYBR Premix EX Taq (Takara Bio, Otsu, Japan). Amplification was carried out in a total volume of 20 μL, containing 2 μL cDNA, 10 μL 2× SYBR Green Premix EX Taq, 7 μL double-distilled H_2_O, and 0.5 μL of each primer. All measurements were performed in triplicate. The relative amount of each studied mRNA was normalized to GAPDH mRNA levels and the data were analyzed according to the 2^−∆∆CT^ method.

### 2.6. Morphological Analysis of Small Intestine

The duodenum, jejunum, and ileum tissues were embedded in paraffin, sectioned (4 μm), stained with hematoxylin and eosin (H&E), and imaged with a Panoramic MIDI slide scanner (3D HISTECH Co., Ltd., Budapest, Hungary). Villus height and crypt depth were measured based on 10 appearance-intact villi and crypt per specimen with Image-pro plus 6.0 (Media Cybernetics, Inc., Bethesda, MD, USA). The villus height and crypt depth refer to the distance from the top of the villus to the crypt opening and the distance from the crypt opening to the base, respectively.

### 2.7. Microbial Genomic DNA Extraction and Quantification

The genomic DNA of microbes in the intestinal contents and mucosa of piglets were extracted with Aidlab Stool DNA kit (Aidlab Biotech, Beijing, China). The qPCR was performed to quantify the total bacteria number using bacteria-specific primer 5′-CGGYCCAGACTCCTACGGG-3′ and primer 5′-TTACCGCGGCTGCTGGCAC-3′ (10 µM) [38], and the total number of *E. faecium* using *E. faecium*-specific primers 5′-TTGAGGCAGACCAGATTGACG-3′ and 5′-CGGAAGTGATGCTTCCTACTG-3′ [39]. The qPCR was carried out in 10 µL reaction mixtures consisting of 1 µL DNA, 0.5 µL of each primer (10 µmol), and 5 µL of universal SYBR qPCR Master Mix (2×) (Accurate Bio-Medical Co., Ltd., Changsha, China). These primers were used to generate amplicon from *Escherichia coli* and *E. faecium* HDRsEf1, respectively, which was subsequently cloned into pMD-18T vector (Takara Bio, Otsu, Japan). The 10-fold serial dilution of plasmid with known concentration was used as a standard for qPCR analysis to determine bacteria copy numbers in samples.

### 2.8. Analysis of Serum Inflammatory Factors

The concentrations of inflammatory factors (including IL-4, IL-6, IL-8, IL-10, IL-12, IFN-γ, TGF-β1) in serum were determined by Quantibody Porcine Cytokine Array kits (RayBiotech, Inc., Atlanta, GA, USA) in accordance with the manufacturer’s protocol.

### 2.9. Statistical Analysis

Differences were determined with Student’s *t*-test by the application of GraphPad Prism 8.0 (GraphPad Software, Inc., San Diego, CA, USA). If there are no additional instructions, our data are shown as mean ± SEM. For all tests, *p*-value *<* 0.05 represents significant differences among groups.

## 3. Results

### 3.1. High-Throughput Sequencing Data

A total of 2,909,872 high-quality sequences were obtained from 60 samples, with an average length of 419 bp. The rarefaction curves tended to be flat, indicating that the current sequencing amount could cover most species in these samples. According to a 97% sequence similarity, these sequences were clustered into 2866 OTUs, and then divided into 45 phyla, 94 classes, 195 orders, 352 families, and 743 genera. 

### 3.2. Effects of HDRsEf1 on Microbial Alpha Diversity in Different Niches

**Figure 1:**
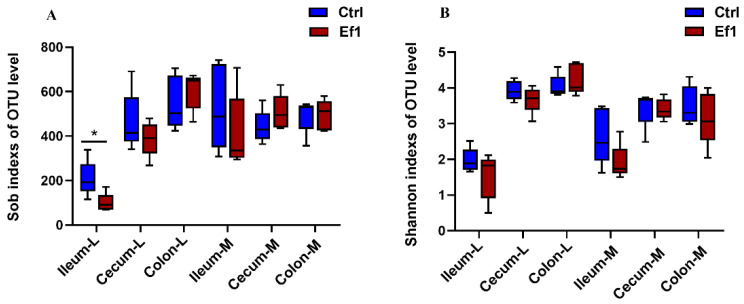
Alpha diversity of gut microbiota in the luminal contents and the corresponding mucosal tissues of ileum, cecum, and colon. (**A**) Sobs index, (**B**) Shannon index. L, luminal contents; M, mucosa; Ctrl, control; Ef1, *Enterococcus faecium* HDRsEf1; n = 5 per group. Values are median ± interquartile range, * *p* < 0.05.

The α diversity indices (sob index and Shannon index) were significantly increased in the colonic luminal contents and cecal luminal contents compared with the ileal luminal contents, while no significant difference of α diversity index was found in the corresponding mucosa (Figure S1A,B). The sob index in ileal luminal contents was significantly decreased by HDRsEf1 administration, while other niches were not affected (Figure 1A,B).

### 3.3. Effects of HDRsEf1 on Total Bacterial Load in Different Gut Niches

**Figure 2:**
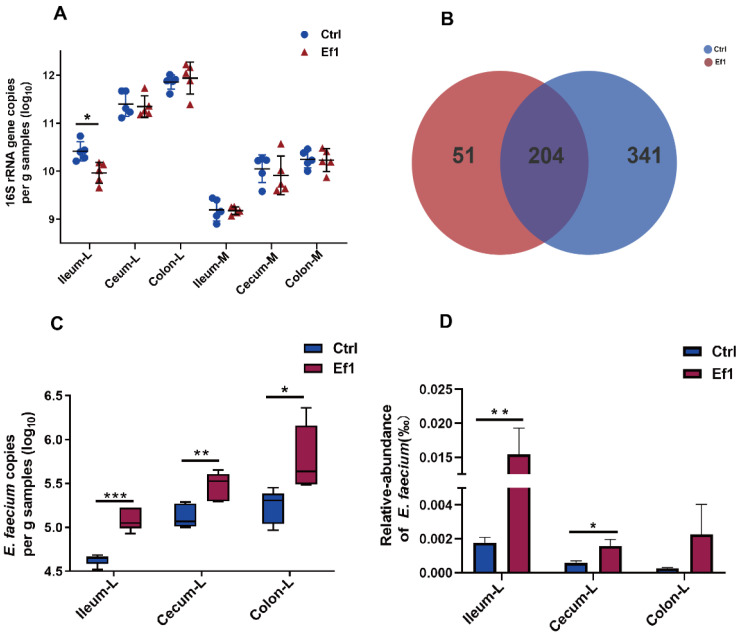
The total bacterial load and *E. faecium* load in different gut niches. (**A**) qPCR determination of total 16S rRNA gene copy number in the mucosal tissues and the corresponding luminal contents of ileum, cecum, and colon. (**B**) Venn diagram for bacterial OTUs number in ileal luminal contents samples. (**C**) qPCR determination for the copy number of *E. faecium* in the luminal contents of ileum, cecum, and colon. (**D**) Relative abundance of *E. faecium.* Data are shown as mean ± SEM and median ± interquartile range, L, luminal contents; M, mucosa; Ctrl, control; Ef1, *E. faecium* HDRsEf1; n = 5 per group. * *p* < 0.05, ** *p* < 0.01, *** *p* < 0.001.

To investigate the impact of HDRsEf1 on the total bacterial load in the intestine, qPCR was used to detect the 16S rRNA gene copy number of bacteria in different niches. It was shown that the total bacterial load increased from the small intestine to the large intestine and from the mucosa to lumen contents (Figure 2A). In addition, the 16S rRNA gene copy number of ileal luminal contents was significantly decreased in the HDRsEf1 group compared with the control group, while there was no significant difference in other niches (Figure 2A). Furthermore, the significantly lower number of 16S rRNA gene copies was confirmed by the decreased number of OTUs, with only 255 OTUs in the HDRsEf1 group while 545 OTUs in the control group identified in ileal luminal contents. (Figure 2B). Taken together, these results indicated that HDRsEf1 could effectively inhibit the growth of microorganisms in ileal luminal contents. In order to verify the colonization of HDRsEf1 in the intestinal tracts, the number of *E. faecium* in intestinal contents was detected with qPCR. The number of *E. faecium* was significantly increased in the ileum, cecum, and colon compared with the control group (Figure 2C), indicating that HDRsEf1 could colonize in the intestine successfully. Moreover, it was shown that *E. faecium* colonizes in the intestine with an extremely low abundance (1/100,000~1/1,000,000) (Figure 2D).

### 3.4. Effects of HDRsEf1 on Microbial Beta Diversity in Different Niches 

**Figure 3:**
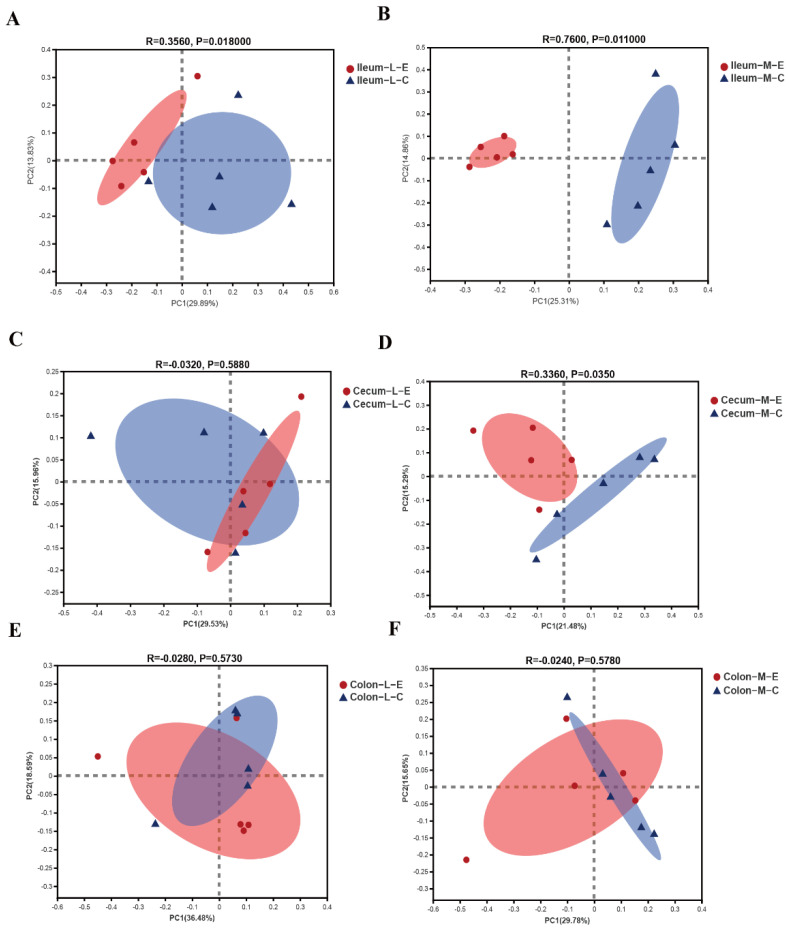
Unweighted principal coordinates (PCoA) of microbiota in different inches. (**A**) Ileal luminal contents. (**B**) Ileal mucosa. (**C**) Cecal luminal contents. (**D**) Cecal mucosa. (**E**) Colonic luminal contents. (**F**) Colonic mucosa. M, mucosa; C, control; E, *Enterococcus faecium* HDRsEf1.

A PCoA plot based upon unweighted principal coordinates (Figure 3) and weighted principal coordinates (Figure S2) was used to analyze the β-diversity of microbiota, an ANOSIM analysis was also used to confirm the difference between the control group and HDRsEf1 group (Table 1). In general, gut microbiota in different gut niches responded differently to HDRsEf1. A clear separation of the microbiota community structure was found between the HDRsEf1 group and the control group in the ileum, especially in the ileal mucosa (Figure 3A,B and Figure S2B). Moreover, cecal microbiota did not separate between different groups except for the mucosal microbiota in the unweighted PCoA plot (Figure 3C,D and Figure S2C,D). In contrast, colonic microbiota, neither from luminal contents nor mucosa, showed separate between the control group and HDRsEf1 group (Figure 3E,F and Figure S2E,F).

### 3.5. Effects of HDRsEf1 on Microbiota Composition in Different Niches

**Figure 4:**
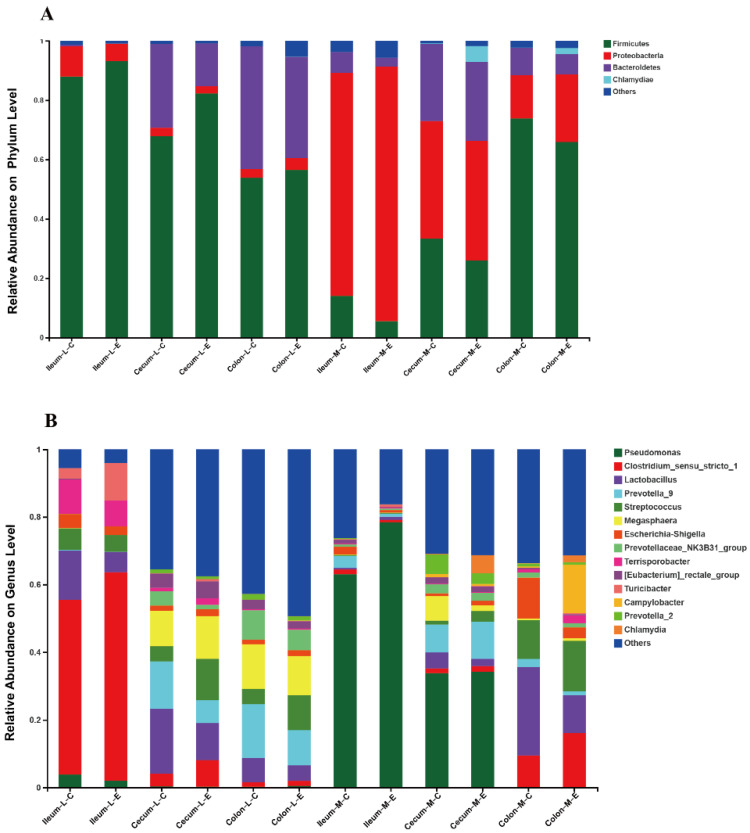
Microbiota composition in different niches. (**A**) Relative abundance of bacterial taxa on the phylum level. (**B**) Relative abundance of bacterial taxa on the genus level. Only phylum with average relative abundance greater than 1% and genus greater than 5% were shown. L, lumen contents; M, mucosa; C, control; E, *Enterococcus faecium* HDRsEf1.

In general, the microbiota across all gut niches were dominated by *Firmicutes*, *Proteobacteria,* and *Bacteroidetes*. At the genus level, the microbiota was mainly composed of *Pseudomonas*, *Escherichia-Shigella* (belonging to *Proteobacteria*), *Lactobacillus*, *Clostridium-sensu-stricto-1*, *Streptococcus* (belonging to *Firmicutes*), and *Prevotella-9*, *prevotella-NK3B31*, *prevotella-2*, *Megasphaera* (belonging to *Bacteroides*). In luminal contents, through the ileum–cecum–colon, the relative abundances of *Firmicutes* and *Proteobacteria* were decreased, while the relative abundance of *Bacteroidetes* increased. In contrast, in the mucosa, the relative abundance of *Firmicutes* increased gradually from the ileum to the cecum and to the colon, while the relative abundance of *Proteobacteria* reduced. In addition, the relative abundance of *Proteobacteria* in the intestinal mucosa was significantly higher than intestinal contents (Figure 4A,B)

**Figure 5:**
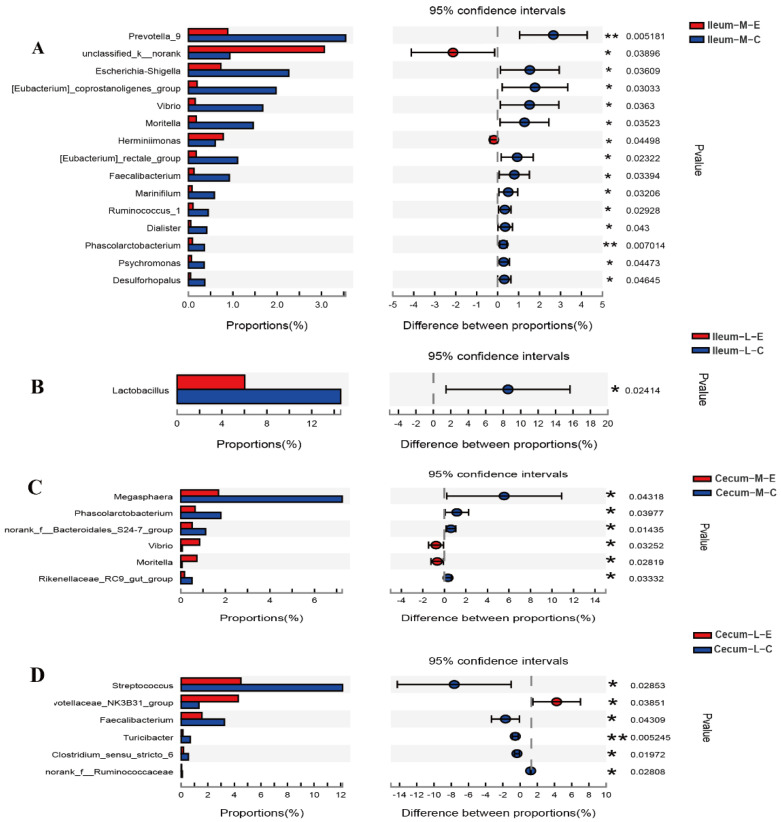
Effects of HDRsEf1 on Microbiota composition in different niches at genus level. (**A**) Ileal mucosal samples at genus level. (**B**) Ileal luminal contents samples at genus level. (**C**) Cecal mucosal samples at genus level. (**D**) Cecal luminal contents samples at genus level. Only relative abundance greater than 0.5% were shown, n = 5 per group, * *p* < 0.05, ** *p* < 0.01.

In this study, it was found that the microbial composition was changed greatly by HDRsEf1 administration. In the ileal mucosa, the relative abundances of 13 genera were significantly decreased (including *Prevotella-9*, *Escherichia-Shigella*, *Vibro*), while the relative abundances of two genera (K- *Norank*, *Herminiimonas*) were significantly increased with HDRsEf1 administration (Figure 5A). In ileal luminal contents, the relative abundance of *Lactobacillus* was significantly decreased with HDRsEf1 administration (Figure 5B). In the cecal mucosa, the relative abundances of four genera (*Megasphaera*, *Phascolarctobacterium*, *norank-f-Bacteroidales-S24-7-group*, *Rikenellaceae-RC9-gut-group*) were significantly decreased in the HDRsEf1 group, while the relative abundances of *Vibrio* and *Moritella* increased (Figure 5C). Importantly, in cecal contents, the relative abundances of five genera (*Streptococcus*, *Faecalibacterium*, *Turcicibacter*, *Clostridium*-*sensu*-*stricto*-6, *Norank*-F-*Ruminococcaceae*), which all belong to *Clostridia*, were significantly increased, while the relative abundance of the *Prevotellaceae*-*NK3B31*-*Group* was significantly decreased with HDRsEf1 administration (Figure 5D).

**Figure 6:**
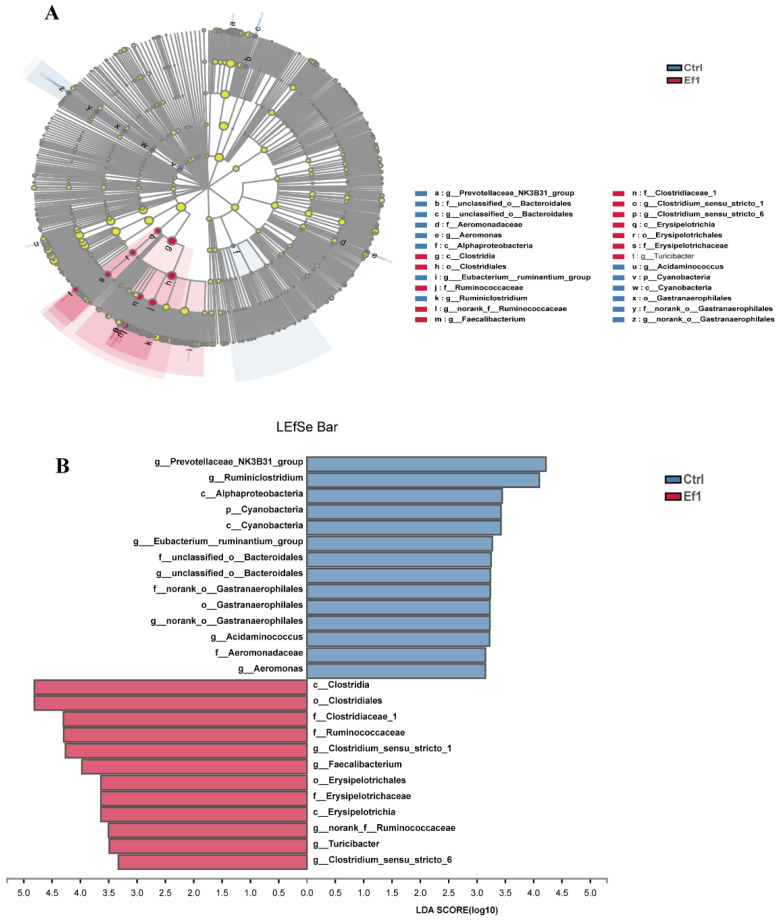
LefSe analysis and LDA score of cecal luminal microbial community from control groups and the HDRsEf1 group. (**A**) Cladogram revealed the microbial taxa with significant differences between the control group and HDRsEf1 group from phyla to genus. The red and blue nodes represent microbial taxa that were enriched in the HDRsEf1 group and the control group, respectively. (**B**) Microbial taxa with LDA score greater than 2.0, the length of the histogram represents the LDA score of the taxa with significant difference. n = 5 per group.

To confirm the difference of intestinal microbiota between the control group and HDRsEf1 group, a Linear discriminant analysis (LDA) and effect size analysis (LefSe) were performed. It was shown that the effects of HDRsEf1 on microbial composition were mainly concentrated in the ileal mucosa and cecal lumen contents. In the ileal mucosa, the relative abundances of numerous taxa were decreased by HDRsEf1 administration, which were mainly enriched in Firmicutes, Bacteroides, and a part of *Proteobacteria* (Figure S3). In cecal luminal contents, the relative abundances of *Clostridia* and *Erysipelotrichia* in the HDRsEf1 group were significantly increased in the HDRsEf1 group. At the genus level, the relative abundances of *Clostridium-sensu-stricto-1*, *Clostridium-sensu-stricto-6*, *Faecalibacterium* (all belong to *Clostridia*), and *Tuncibacter* (belong to *Erysipelotrichia*) were elevated in the HDRsEf1 group while the relative abundances of *Prevotellaceae*-*NK3B31*-*Group*, *unclassed*-o-*bacteroidales,* and *norank*-o-*Gastranaerophilales* were increased in the control group (Figure 6).

### 3.6. Effects of HDRsEf1 on Intestinal Inflammation

**Figure 7:**
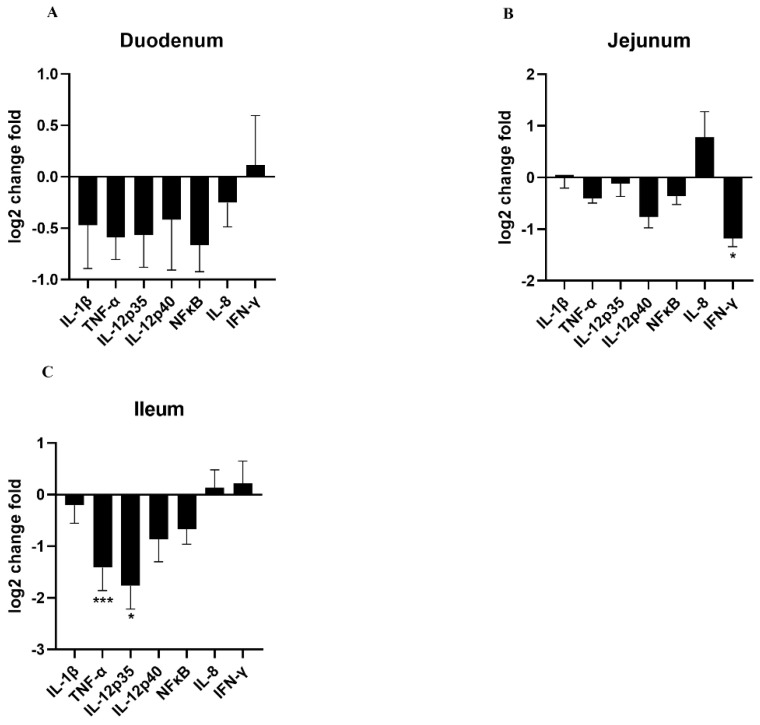
Effects of HDRsEf1 on inflammatory responses in small intestine. (**A**–**C**) Inflammatory factors in mucosa of duodenum, jejunum, and ileum. n = 5, * *p* < 0.05, *** *p* < 0.001.

**Figure 8:**
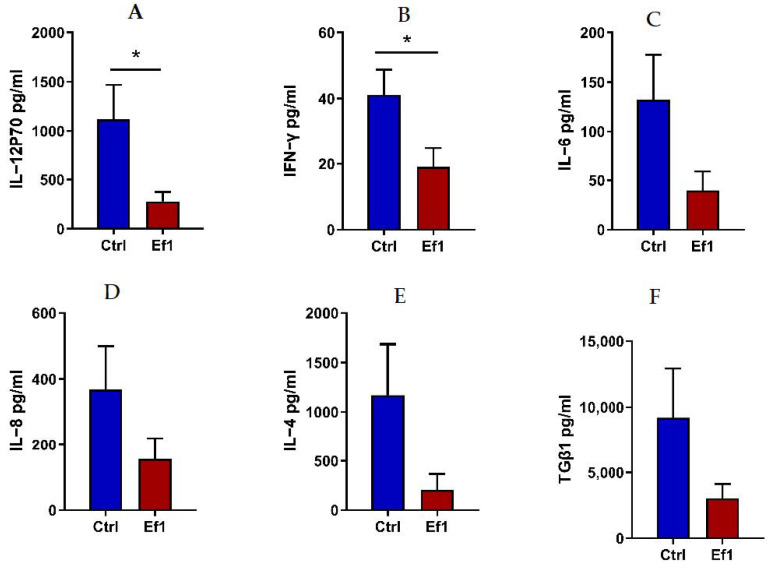
Effects of HDRsEf1 on inflammatory responses in serum. (**A**–**F**) The levels of inflammatory factors in serum, including IL-12, IFN-γ, IL-6, IL-8, IL-4, and TGF-β1. Ctrl, control; Ef1, *Enterococcus faecium* HDRsEf1. Data are represented as mean ± SEM, n = 5, * *p* < 0.05.

It is well known that weaning stress could cause excessive intestinal inflammation, which could contribute to intestinal injury and diarrhea. To understand the anti-inflammatory effect of HDRsEf1 on piglets, the inflammatory factors in the mucosa of the small intestine and serum were measured and the morphology of the small intestine was analyzed. In this study, the expression of TNF-α, IL-12p35 in the ileum and IFN-γ in the duodenum significantly decreased in the HDRsEf1 group compared with the control group (Figure 7A–C). Moreover, in agreement with intestinal samples, piglets in the HDRsEf1 group exerted a significantly lower expression of IL-12 and IFN-γ in serum samples compared with the control group (Figure 8A,B). Moreover, HDRsEf1 significantly increased the villus height in the jejunum and the villus height to the crypt depth ratio in duodenal (Figure S4A–D). Taken together, these results indicated that HDRsEf1 could effectively alleviate inflammatory responses and improved the intestine morphology of piglets.

### 3.7. Correlation Analysis of Gut Microbiota Alterations and Inflammatory Responses

**Figure 9:**
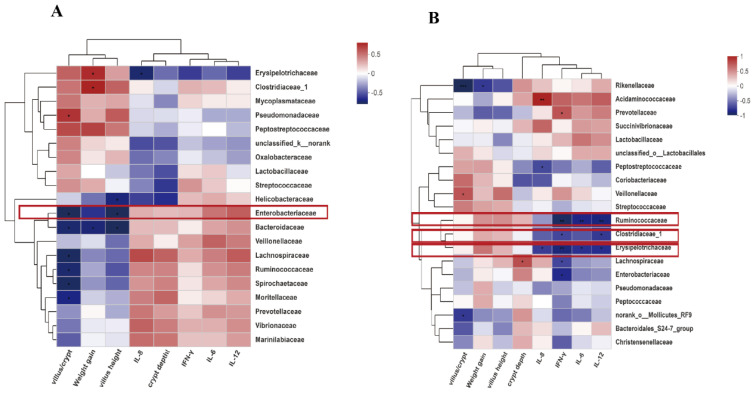
Heatmap for correlation analysis between microbiota inflammatory responses or villus height in ileal mucosa and cecal luminal contents. (**A**) Microbiota in ileal mucosa at family level. (**B**) Microbiota in cecal luminal contents at family level. Red box, microorganisms with significant difference between HDRsEf1 group and control group. * *p* < 0.05, ** *p* < 0.01, *** *p* < 0.001.

To further investigate whether the changes of inflammatory responses were correlated with the change of gut microorganisms, we studied the correlation between inflammatory cytokine expression, the height of intestinal villi, daily body weight gain, and gut microorganisms that were significantly affected by HDRsEf1 from the phylum to genus level. The correlation analysis showed that the relative abundances of *Enterobacteriaceae* and *Escherichia-Shigella* in the ileum mucosa were significantly negatively correlated with the villus height of the small intestine (Figure 9A and Figure S5A). The relative abundances of *Clostridiaceae-1*, *Rumencoccidae,* and *Erysipelotrichaceae* in cecum contents were significantly negatively correlated with the expression of inflammatory factors (Figure 9B). In *Clostridiaceae-1*, the relative abundances of *Clostridium-sensu-stricto-1* were significantly negatively correlated with inflammatory factors IL-12, IL-6 and IFN-γ in serum and the relative abundance of *Faecalibacterium* was significantly positively correlated with body weight and villus height (Figure S5B). However, there was no correlative relationships between gut microorganism’s alterations and inflammatory responses in other inches.

## 4. Discussion

Post-weaning diarrhea in piglets frequently causes serious complications and is associated with enteric infections due to the overuse of antibiotics [1]. Probiotics supplements have promising effects on gut microbiota and have been commonly fed to improve the growth performance of weaned piglets [4,40]. Previous studies have shown that HDRsEf1 could significantly reduce the incidence of diarrhea of piglets [6]. However, the effects of HDRsEf1 on gut microbiota in weaned piglets are poorly understood. In this study, we investigated the effects of HDRsEf1 on gut microbiota in six different ecological niches: luminal contents and the mucosa of the ileum, cecum, and middle colon. In addition, we analyzed the effects of HDRsEf1 on intestinal development and inflammatory responses in piglets. It was shown that the use of HDRsEf1 significantly altered the composition of microbiota in the ileum and cecum. At the same time, these changes of gut microbiota were correlated with the improvement of intestinal development and the suppression of inflammation.

Many studies have shown that probiotics could play a beneficial role by affecting gut microbiota [20,41]. In this study, the utilization of HDRsEf1 significantly reduced the α diversity and total bacterial load in ileum contents. These results demonstrated for the first time that *Enterococcus faecium* could decrease the number of microorganisms in the small intestine of piglets. Probably, these effects can be attributed to the ability of *E. faecium* to secrete bacteriocin, organic acid, and other bactericidal substances [42]. Nutrients are mainly absorbed in the small intestine, and microorganisms in small intestine, especially ileum, are competitive with the host in nutrient absorption [43,44]. It has been proposed that the reduction in the microbial competition for nutrients and harmful microbial metabolites in the small intestine is the primary mechanism by which antibiotics improve animal performance [18,43]. HDRsEf1 plays a role such as antibiotics in inhibiting the growth of small intestinal microorganisms, which might be one of the mechanisms of HDRsEf1 that improved the performance of piglets. 

The analysis of PCoA and ANOSIM further revealed that HDRsEf1 significantly affected the microbiota community structure of the ileum, while it had no significant effect on the colon. Probably, it was due to the higher diversity of cecal and colonic microbiota, which cumulatively contributed to the stability of the microbiota community. In addition, it was found that the relative abundance of *E. faecium* in the intestine was extremely low (1/100,000~1/1,000,000), which indicated, again, that *E. faecium* played a role by regulating gut microbiota. 

In this study, the relative abundance of *Escherichia-shigella* in the ileal mucosa was reduced with the utilization of HDRsEf1, which was consistent with previous studies that *E. faecium* could alleviate the infection caused by pathogenic *Escherichia coli* [45,46]. In pig breeding, *Escherichia coli* is an important opportunistic pathogen in newborn and weaned piglets, which can cause diarrhea, dehydration, growth retardation, and death of piglets [47,48,49,50]. In addition, due to the abuse of antibiotics, the drug resistance of *Escherichia coli* is seriously increasing, which has posed a serious threat to the breeding industry and public health [51]. Some strains of *Escherichia coli*, such as enterotoxin-producing *Escherichia coli* (ETEC), could adhere to specific receptors of the ileal mucosa, damage the intestinal barrier, decrease the intestinal villi height and change the intestinal morphology [52,53]. Our data were coincident with these results. The utilization of HDRsEf1 enhanced the height of intestinal villi and reduced inflammatory factors in the intestinal mucosa. The increased intestinal villous height was negatively correlated with the relative abundance of *Escherichia-Shigell*a in the ileal mucosa, indicating that *E. faecium* could improve the intestinal development and growth of piglets by inhibiting the growth of *Escherichia-shigella.*

In cecal luminal contents, the microorganisms in *Clostridiales* in the HDRsEf1 group were significantly increased, including *Clotridiaceae-1* and *Ruminococcaceae* in the family level and *Clotridium-sensu-stricto-6*, *Clotridium-sensu-stricto-1*, and *Faeccalibacterium* in the genus level, which all are short-chained fatty acids (SCFA) producing taxa. It has been reported that *Clostridium* plays a key role in butyrate production, by which it can induce the production of Treg CD4^+^ T cells and play an anti-inflammatory role [54,55]. Some studies also reported that *Faecalibacterium prausnitzii* could attenuate the activation of the inflammatory pathway by producing butyrate, which could maintain the intestinal health [56]. The weaning piglets were often accompanied by an excessive inflammatory response, which might lead to both epithelial barrier dysfunction and diarrhea [57]. In this study, HDRsEf1 decreased the expression of IL-12p70 and IFN-γ in serum. Through a correlation analysis, the elevated relative abundances of *Clostridiaceae-1*, *Rumencoccidae*, *Erysipelotrichaceae,* and *Clotridium-sensu-stricto-1* in cecum contents were negatively correlated with the level of inflammatory factors IL-12, IL-6 and IFN-γ. The enhanced abundance of *Faecalibactrium* was positively correlated with body weight gain and villus height. A possible reason is that these taxa induced an immune tolerance and promoted the intestinal health through the production of butyrate. Although HDRsEf1 alone did not produce SCFAs, HDRsEf1 expanded intestinal butyrate-producing bacteria, which indicated that HDRsEf1 indirectly increased the production of SCFAs and down regulated inflammation. In the next step, we would explore the effects of HDRsEf1 on gut microbiota from a metabolism perspective and verify our results by germ-free animals.

In summary, this experiment provided the evidence that *E. faecium* HDRsEf1 could effectively alter gut microbiota and the alteration in different niches of intestine was obviously different, thereby alleviating inflammation and improving the intestinal morphological development in weaned piglets. The response of microbiota in the foregut and cecum has been rarely studied in either humans or animals. To fully unveil the response of intestinal microbiota to probiotics, the research on microbiota in different intestine segments is more recommended. Taken together, this study provided an insight into the mechanism of *E. faecium* in piglets and provided a reference for probiotics for humans and animals.

## Figures and Tables

**Table 1:** ANOSIM R-values of microbial community between HDRsEf1 group and the control group.

Variables	Unweighted (R)	*p*-Value	Weighted (R)	*p*-Value
C VS E				
Ileal Lumen	0.3560	0.018	0,024	0.348
Ileal Mucosa	0.7600	0.011	0.4600	0.014
Cecal Lumen	−0.0320	0.588	0.3160	0.068
Cecal Mucosa	0.336	0.035	−0.052	0.430
Colonic Lumen	−0.028	0.573	0.0760	0.691
Colonic Mucosa	−0.024	0.578	0.000	0.793

## Data Availability

The sequences used in this manuscript were submitted to NCBI-SRA under the BioProject accession number PRJNA694358.

## Supplementary Materials

The following are available online at https://www.mdpi.com/article/10.3390/microorganisms9081767/s1, Figure S1: Alpha diversity of microbiota in different niches of piglets. (A) Sobs index, (B) Shannon index. L, luminal contents; M, mucosa, Figure S2: weighted principal coordinates (PCoA) of microbiota in different inches. (A) Ileal luminal contents. (B) Ileal mucosa. (C) Cecal luminal contents. (D) Cecal mucosa. (E) Colonic luminal contents. (F) Colonic mucosa. M, mucosa; C, control; E, *Enterococcus faecium* HDRsEf1, Figure S3: LefSe analysis and LDA score of microbial community in ileal mucosa from control groups and HDRsEf1 group. (A) Cladogram revealed the microbial taxa with significant differences between the control group and HDRsEf1 group from phyla to genus. The red and blue nodes represent microbial taxa that were enriched in the HDRsEf1 group and the control group, respectively. (B) Microbial taxa with LDA score greater than 2.0, the length of the histogram represents the LDA score of the taxa with significant difference. n = 5 per group, Figure S4: effects of HDRsEf1 on intestinal morphology. (A) The morphology of duodenum, jejunum, and ileum, respectively. (B–D) Statistical analysis of villus height (µm), crypt depth (µm), and the ratios of villus height (µm) to crypt depth (µm) in duodenum, jejunum, and ileum of weaned piglets. Ctrl, control; Ef1, HDRsEf1. Data are represented as mean ± SEM and median ± interquartile range, n = 5, * *p* < 0.05, Figure S5: heatmap for correlation analysis between microbiota and villus height or inflammatory responses in ileum mucosa and cecal luminal contents. (A) Microbiota in ileum mucosa at genus level. (B) Microbiota in cecal luminal contents at genus level. Red box, microorganisms with significant difference between HDRsEf1 group and control group. * *p* < 0.05, ** *p* < 0.01, Table S1: the primer sequences for RT-qPCR.
