# Supplementary material for: Different Responses of Microbiota across Intestinal Tract to Enterococcus faecium HDRsEf1 and Their Correlation with Inflammation in Weaned Piglets
Source: Microorganisms. 2021 Aug 19;9(8):1767. doi: 10.3390/microorganisms9081767 (PMC8402050; doi:10.3390/microorganisms9081767)
Supplement: Supplementary file 1 [file microorganisms-09-01767-s001.zip › supplementary materials/Table S1.pdf]

Genes	Primer sequences (5’-3’)	Fragment size(bps)
IL-1 β	F:GCTGGAGGATATAGACCCC<br>R:GTTGGGGTACAGGGCAGAC	115
TNF α	F:TTCCAGCTGGCCCCTTGAGC<br>R:GAGGGCATTGGCATACCCAC	146
IFN-γ	F:GCCATTCAAAGGAGCATGGA<br>R:TTCACTGATGGCTTTGCGCT	144
GAPDH	F:GAAGGTCGGAGTGAACGGAT<br>R:CATGGGTAGAATCATACTGGAACA	149
IL-12p35	F:ACCACCTGGACCATCAT<br>R:GGGTTTGTTTGGCCTTCT	135
IL-12p40	F:ATCCTCCCAAGAATCTGC<br>R:TGCCCTGAACTTGAACAC	142
NFκB	F: GGCTATAACTCGCTTGGTGACAGG<br>R: CCGCAATGGAGGAGAAGTCTTCG	
IL-8	F AGAACTTCGATGCCAGTGC<br>R GGCAGACCTCTTTTCCATTG	143
