# Supplementary figures and images for: Different Responses of Microbiota across Intestinal Tract to Enterococcus faecium HDRsEf1 and Their Correlation with Inflammation in Weaned Piglets
Source: Microorganisms. 2021 Aug 19;9(8):1767. doi: 10.3390/microorganisms9081767 (PMC8402050; doi:10.3390/microorganisms9081767)

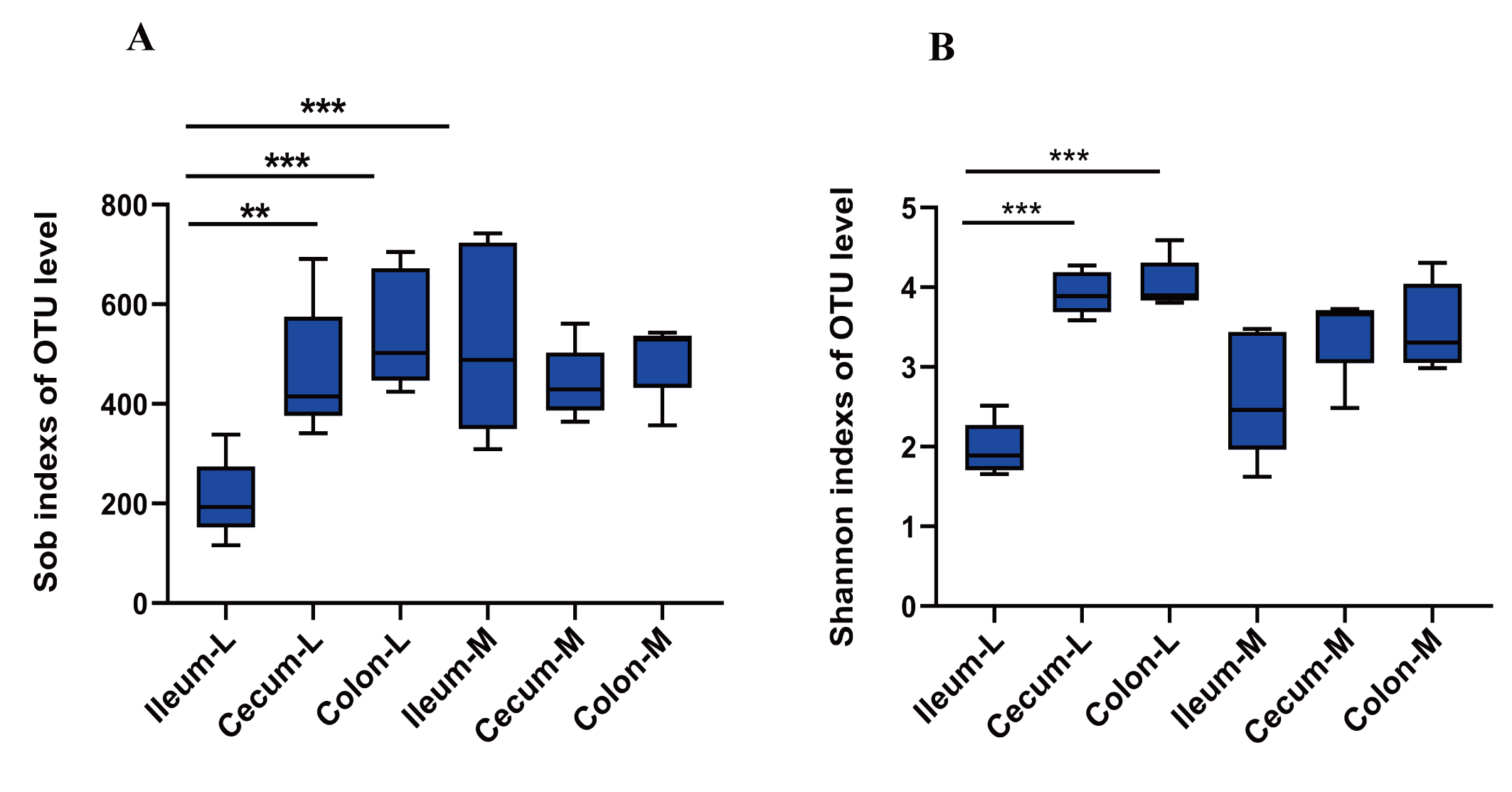

Supplement: Supplementary file 1 [file microorganisms-09-01767-s001.zip › supplementary materials/FigureS1.tif]

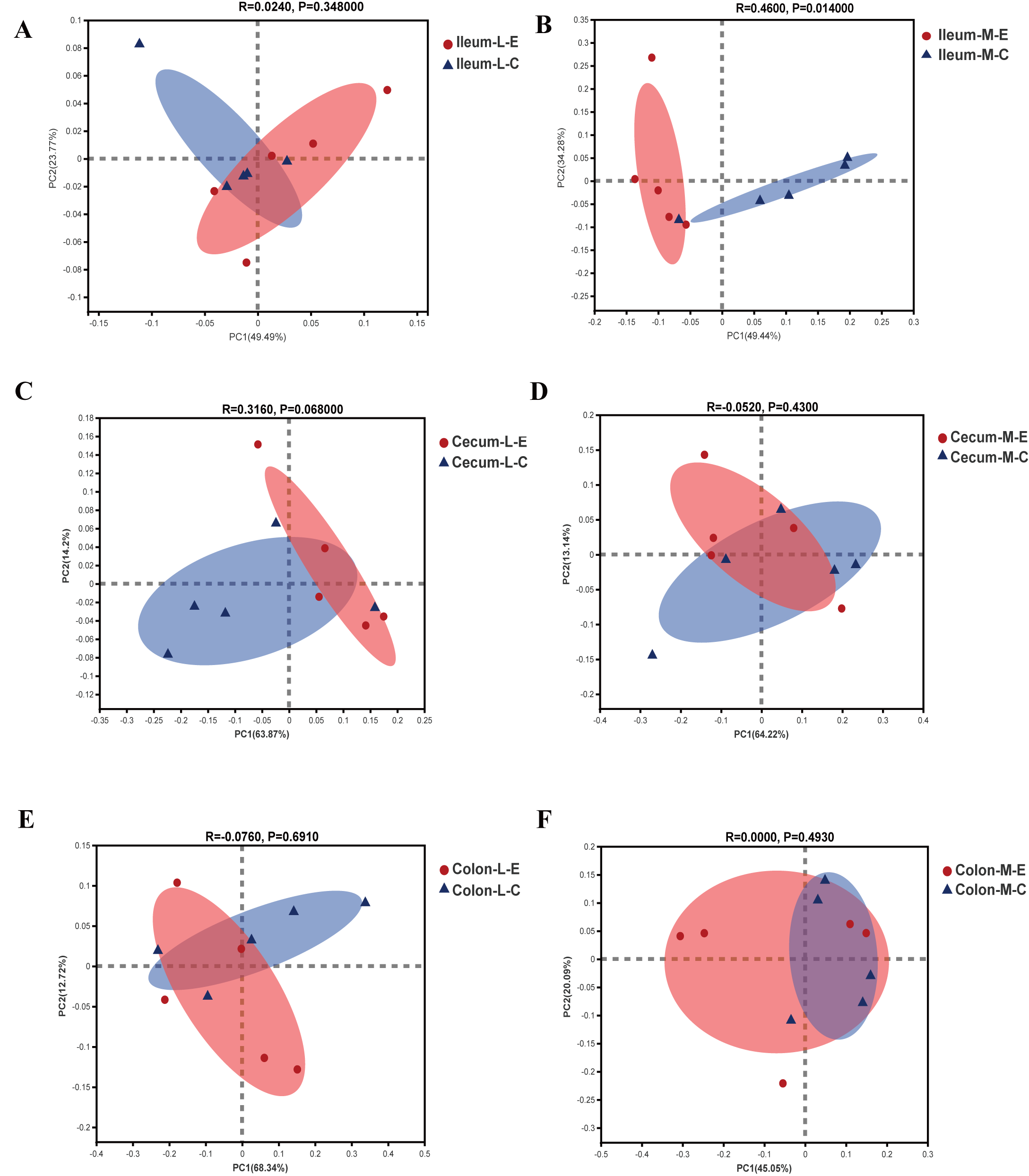

Supplement: Supplementary file 1 [file microorganisms-09-01767-s001.zip › supplementary materials/FigureS2.tif]

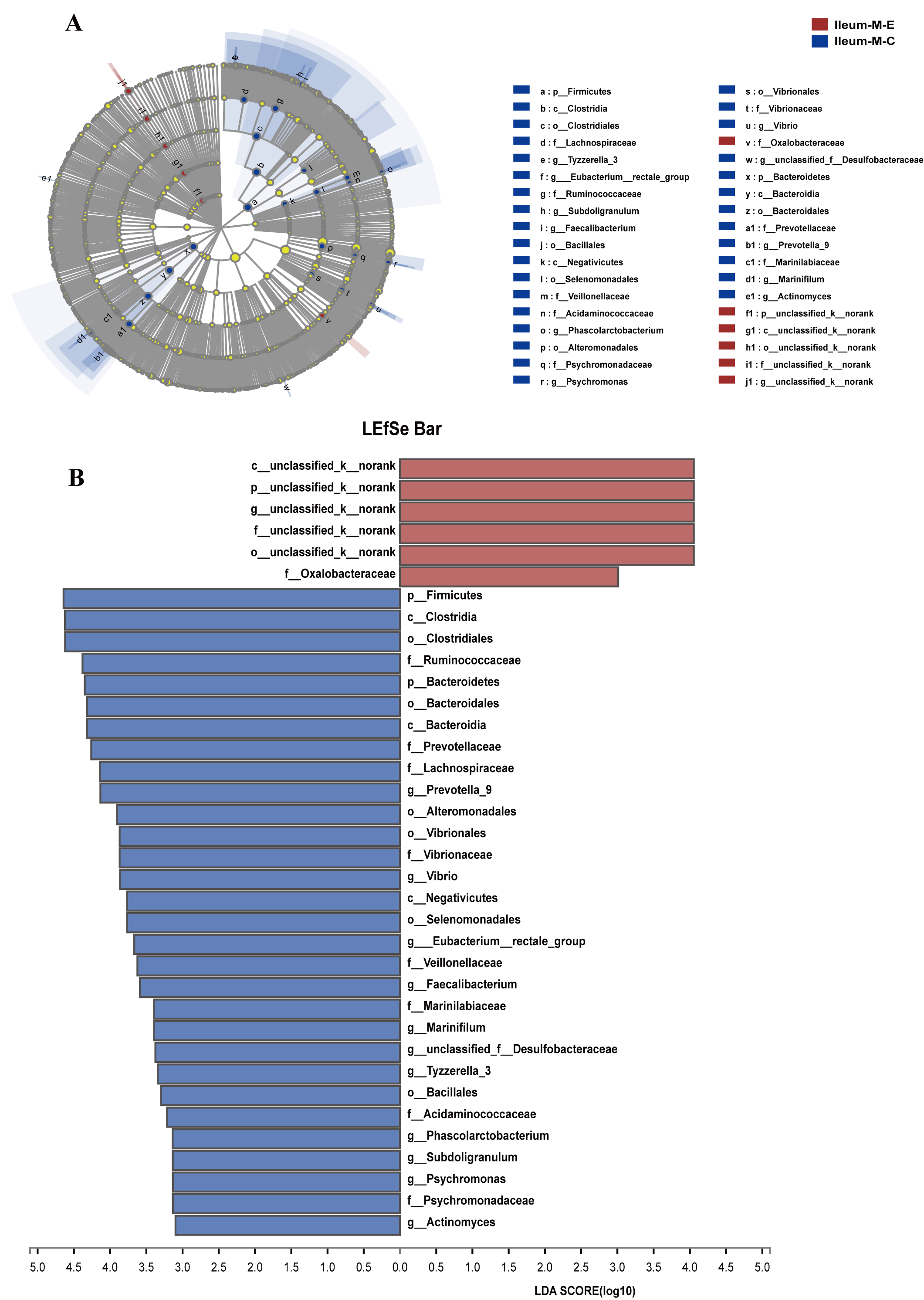

Supplement: Supplementary file 1 [file microorganisms-09-01767-s001.zip › supplementary materials/FigureS3.tif]

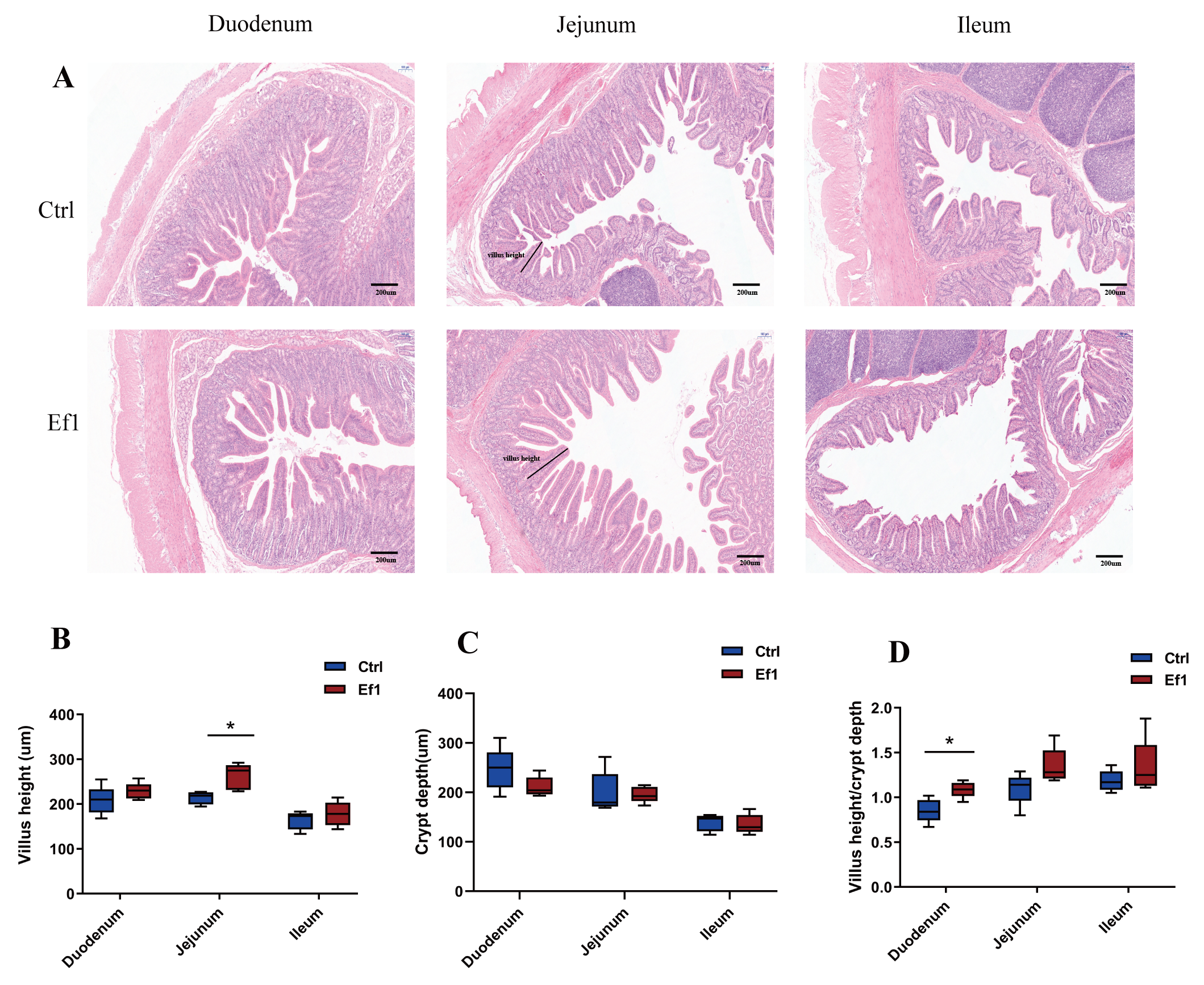

Supplement: Supplementary file 1 [file microorganisms-09-01767-s001.zip › supplementary materials/FigureS4.tif]

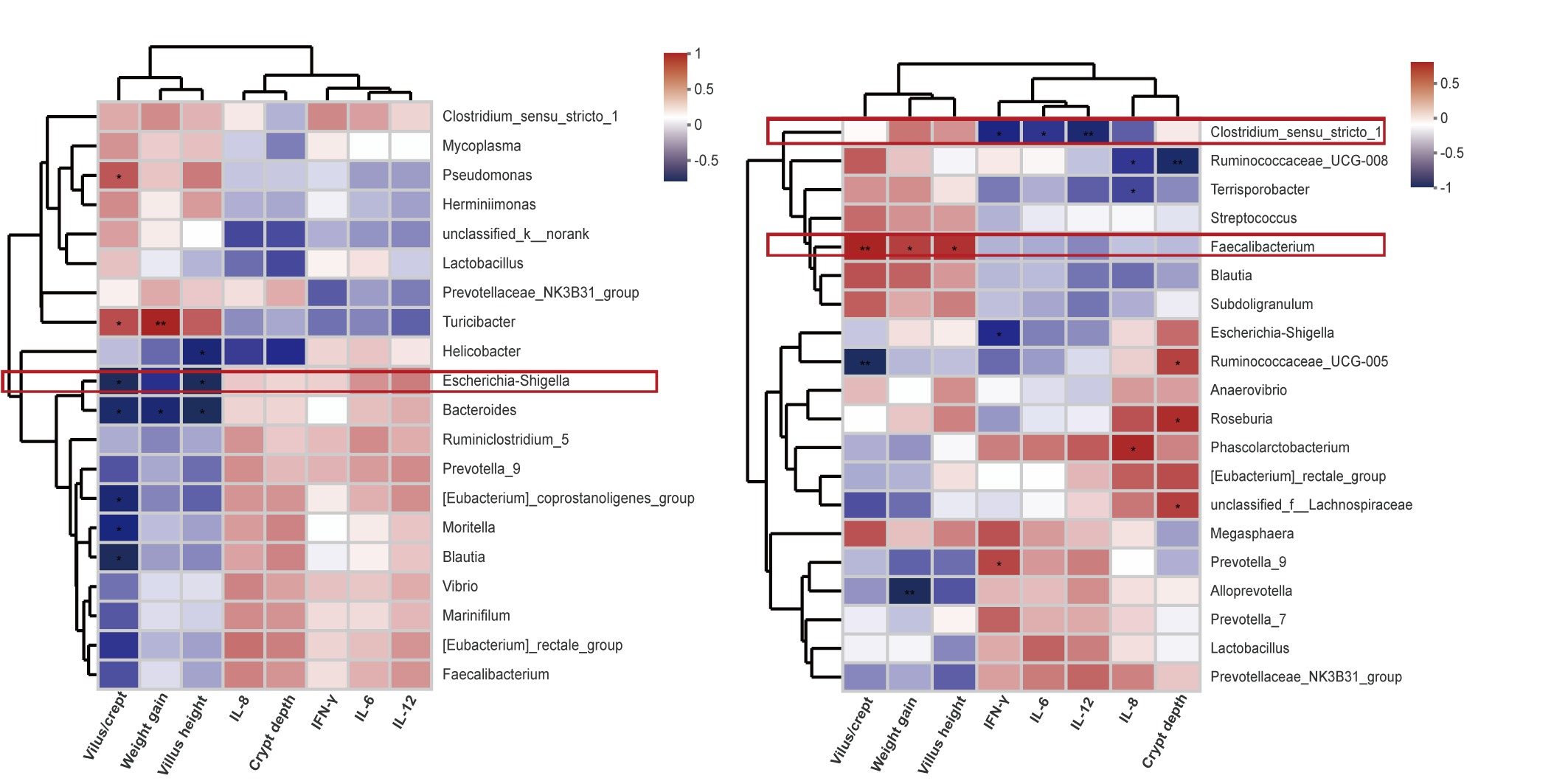

Supplement: Supplementary file 1 [file microorganisms-09-01767-s001.zip › supplementary materials/FigureS5.tif]
